# Risk Factors of COVID-19 Severity and Related Death in Children in the Post-Pandemic Era

**DOI:** 10.3390/microorganisms14091984

**Published:** 2026-09-08

**Authors:** Laura G. Coelho, Lilian M. Diniz, Stella C. Galante, Cristiane S. Dias, Maria Christina L. Oliveira, Enrico A. Colosimo, Ana Cristina Simões e Silva, Fernanda N. Duelis, Maria Eduarda T. Bernardes, Julia O. Zavitoski, Daniella R. B. Martelli, Fabrício Emanuel S. Oliveira, Hercílio Martelli-Júnior, Adriano L. Santos, Robert H. Mak, Eduardo A. Oliveira

**Affiliations:** 1 Department of Pediatrics, Health Sciences Postgraduate Program, School of Medicine, University Federal of Minas Gerais (UFMG), Belo Horizonte 31310-100, MG, Brazil; 2 Department of Statistics, Federal University of Minas Gerais (UFMG), Belo Horizonte 31270-901, MG, Brazil; 3 Health Science/Primary Care Postgraduate Program, State University of Montes Claros (Unimontes), Montes Claros 39401-089, MG, Brazil; 4 Department of Engineering and Informatics, Federal Institute of Science and Technology of Minas Gerais (IFMG), Belo Horizonte 30575-180, MG, Brazil; 5 Department of Pediatrics, Division of Pediatric Nephrology, University of California, San Diego, CA 92093, USA

**Keywords:** COVID-19, children, comorbidities, vaccine, post-pandemic, mortality

## Abstract

In the post-pandemic era, identifying children who are most susceptible to severe illness and COVID-19-related mortality is essential for guiding public health policies. This study examined the risk factors for COVID-19-related severe illness and mortality from 2023 to mid-2025. We conducted a population-based cohort study using nationwide Brazilian data from patients aged <18 years with laboratory-confirmed SARS-CoV-2 infection between January 2023 and June 2025. The primary outcomes were COVID-19-related severity and death. Separate binary multivariable logistic regression models were developed for each of the outcomes. Among 465,689 children, 1.3% (n = 5963) developed severe illness, and 0.18% (n = 847) died. Factors associated with an increased risk of severe illness included age < 2 years, presence of comorbidities, Indigenous ethnicity, and lack of vaccination. Neurological disorders conferred the highest risk among the clinical conditions (adjusted odds ratio [aOR] = 34.1; 95% CI: 27.9–41.8). Regional differences were also observed; the North and Northeast regions showed higher mortality (aOR = 2.3; 95% CI: 1.8–3.0) than the Central-West region. Compared with White ethnicity, non-White ethnicities had higher mortality: Indigenous (aOR = 23.5; 95% CI: 13.5–39.3), Black (aOR = 1.95; 95% CI: 1.29–2.92), and Brown (aOR = 1.43; 95% CI: 1.18–1.73) ethnicities. Lack of any vaccine dose was associated with a significantly increased risk of severe illness (aOR = 1.39; 95% CI: 1.15–1.67. *p* < 0.001) and death (aOR = 2.1; 95% CI: 1.3–3.5; *p* < 0.001). In the post-pandemic era, younger age, comorbidities, sociodemographic disparities, and lack of vaccination were associated with an increased risk of severe illness and COVID-19-related death in the pediatric population.

## 1. Introduction

Understanding of SARS-CoV-2 infection in pediatric populations has significantly changed from the early days of the pandemic to the present post-pandemic period [1]. During the pandemic, most children experienced mild infections, and severe outcomes were rare, particularly among healthy children [2,3,4]. However, children with comorbidities are consistently identified as having an increased risk of complications [5].

However, further studies have revealed a more complex picture, particularly regarding global inequalities. In a previous study, we showed increased mortality among children hospitalized with COVID-19 in Brazil, which has been supported by data from other low- and middle-income countries [6,7]. These data suggest that the most severe consequences for children are related to a confluence of social determinants of health (SDOH) and pre-existing comorbidities [8,9,10,11]. Previously, we reported findings from the pandemic period in Brazil of 2.8 million pediatric patients, showing that the overall mortality rate was low (0.17%) but increased to 7.9% among hospitalized patients, and 44.3% of the children who died did not receive full critical care. Regional differences were evident, with higher case fatality rates in the North (13.02%) and Northeast (11.6%) regions of Brazil and among Indigenous populations (22.54%), highlighting the significant influence of social inequalities on COVID-19 mortality [11].

On 5 May 2023, the World Health Organization (WHO) announced the end of the global COVID-19 public health emergency. The immunological landscape has changed after the pandemic owing to global vaccination campaigns and naturally acquired immunity in the population [12]. The transition from a predominantly susceptible population to one with substantial vaccine-induced and infection-derived immunity has considerably modified the clinical and public health consequences of COVID-19, despite the continued emergence of viral variants with immune-evasive properties [13,14,15]. In 2023, it was estimated that over 96% of children in the United States had been exposed to SARS-CoV-2, indicating significant penetration of the virus in the pediatric population [1]. Therefore, concerns remain about the changing immunological environment, emergence of new variants, and long-term effects [16]. The post-COVID-19 sequelae, such as multisystem inflammatory syndrome in children (MIS-C) and long COVID, have become important problems, with estimates that 2–10% of pediatric infected patients might develop long COVID, affecting their quality of life and daily activities [16,17,18,19,20,21,22].

However, despite this new scenario, few studies have explored the risk determinants of severe COVID-19 in children and adolescents after the end of the pandemic. We found a single U.S. study that showed that a small percentage of pediatric patients still experience severe COVID-19 [23]. Notably, in this cohort, only a small percentage of hospitalized children were fully vaccinated, highlighting the continued vulnerability of under-immunized populations. Similarly, there are limited data on vaccine effectiveness following the end of the pandemic emergency. Vaccination has a protective effect, but its efficacy varies depending on the variant and decreases over time [20]. In a recent study, the updated vaccines for 2024–2025 provided substantial protection against emergency department or urgent care visits related to COVID: 76% effectiveness in children aged 9 months to 4 years and 56% in children aged 5–17 years [24].

In the current context, identifying pediatric patients at high risk of severe COVID-19 is important to guide public health approaches, including the target populations for booster vaccinations. The primary objective of this study was to identify demographic, clinical, and vaccination-related risk factors for COVID-19-related severity and death among Brazilian children and adolescents during the post-pandemic period (2023–2025).

## 2. Materials and Methods

### 2.1. Study Design, Participants, Study Period, and Data Sources

**Figure 1:**
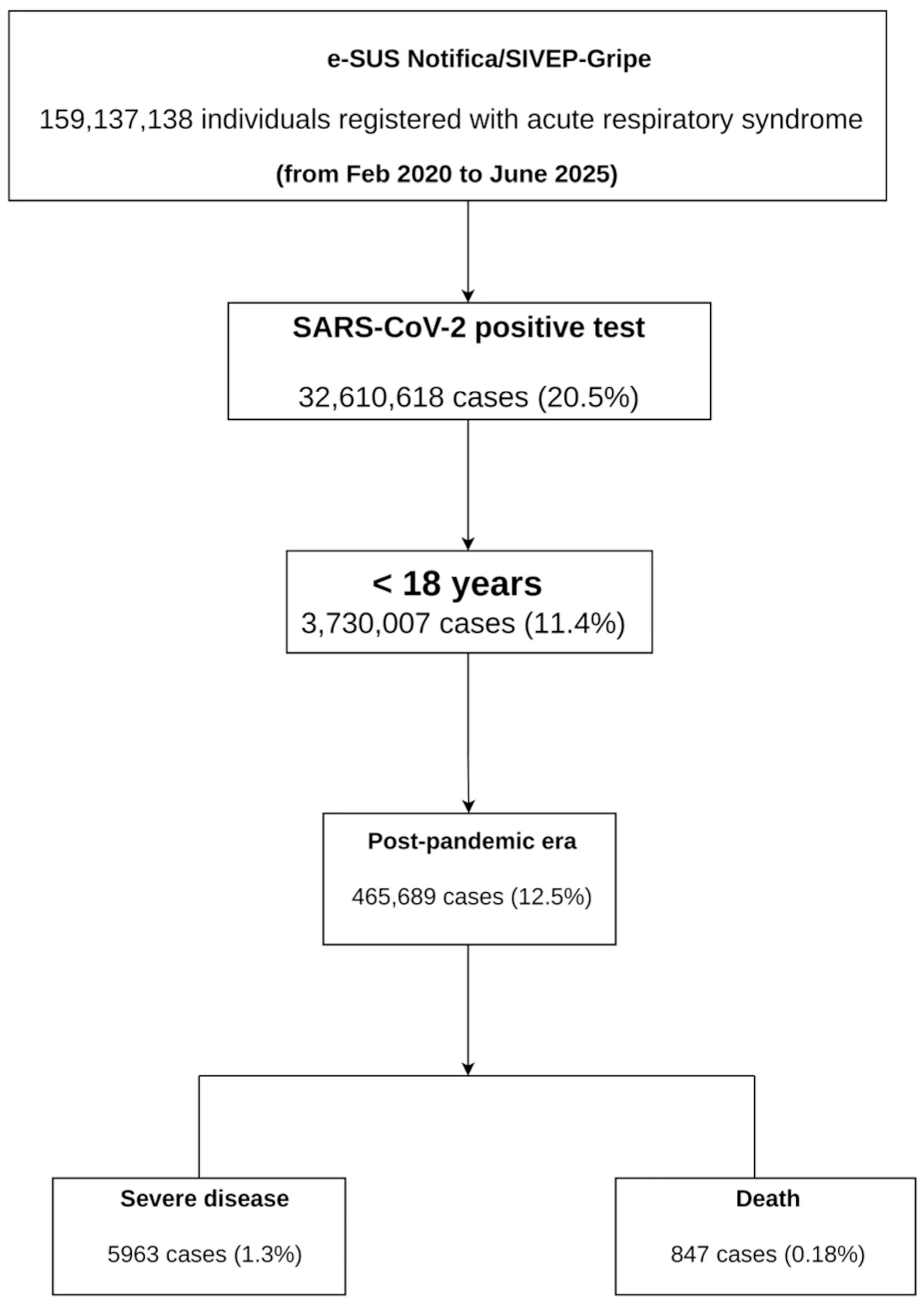
Flowchart of the selected cohort included in the study.

We conducted a retrospective cohort study utilizing population data derived from two official Brazilian national COVID-19 surveillance systems provided by the Ministry of Health: (1) e-SUS Notifica, which tracks non-hospitalized individuals, and (2) SIVEP-Gripe, which monitors hospitalized patients. The criteria included age < 18 years, laboratory-confirmed SARS-CoV-2 infection, and cases registered in these systems during the post-pandemic period, defined for this study as cases registered between 1 January 2023 and 30 June 2025. The exclusion criteria included duplicate records and records with missing key identifiers. Detailed information on both systems is available at https://dados.gov.br/dados/conjuntos-dados/srag-2021-e-2022 (accessed on 10 July 2025). The selection process for the cases included in our analysis from these comprehensive data sources is depicted in the flowchart (Figure 1).

### 2.2. Predictive Factors

The predictive factors included in the analysis were demographic data, clinical data, and vaccination status. The collected demographic data included age, sex, ethnicity, and geographic regions. Age was categorized into four groups: <2 years, 2–4 years, 5–11 years, and 12–17 years. Ethnicity was classified into five groups according to the Brazilian Institute of Geography and Statistics (IBGE) system: White, Black, Brown (mixed race, Pardo in Portuguese), Asian, and Indigenous [25]. The geographic regions were categorized into five official Brazilian macro-regions (Southeast, South, Central-West, Northeast, and North). The clinical data collected included the date of COVID-19 symptom onset, date of hospital admission, presenting signs and symptoms, and the presence of comorbidities. We identified comorbidities using specific database fields for pre-existing chronic conditions, including asthma, diabetes, obesity, immunodeficiency, malignancies, and cardiac, pulmonary, renal, neurological, and hematological diseases. For analysis, we categorized comorbidity status as a dichotomous variable (present/absent) and the number of conditions (none, one, or two or more). Given the nature of national surveillance databases, in which the absence of a recorded condition is likely to indicate a true absence, we handled missing data in the comorbidity fields by imputing absence. This conservative approach was consistent with previous studies that used similar data sources [7,25]. Vaccination status was determined based on the number of vaccine doses administered at least 14 days before the onset of SARS-CoV-2 infection symptoms, which is the requisite period for an immune response to develop. Individuals were classified into four categories: (1) unvaccinated, (2) one dose, (3) two doses, and (4) three or more doses. Details regarding the management of misclassifications in vaccination status and missing vaccination dates are provided elsewhere [26]. The neurological disease field does not include diagnostic codes or a predefined list of conditions; therefore, the specific disorders included in this category could not be characterized further. Asthma severity was not available, and asthma/chronic pulmonary disease was analyzed as a combined reporting category.

During the study period (January 2023–June 2025), pediatric COVID-19 vaccination in Brazil relied mainly on two platforms authorized by the National Health Surveillance Agency (Anvisa) and offered through the National Immunization Program (PNI): pediatric Comirnaty (Pfizer-BioNTech, mRNA) and CoronaVac (Butantan/Sinovac, inactivated virus). Comirnaty was authorized for ages 12–17 years in June 2021, ages 5–11 years in December 2021, and ages 6 months-4 years in September 2022; the youngest group entered the routine national childhood immunization calendar on 1 January 2024. CoronaVac was authorized for ages 6–17 years in January 2022 and for ages 3–5 years in July 2022. The chronology of the rollout of Brazilian vaccines for children and adolescents during the pandemic has been provided in detail elsewhere [27]. Thus, all age strata used in this study had at least partial vaccine eligibility during the study period, although infants younger than 6 months remained ineligible.

**Figure 2:**
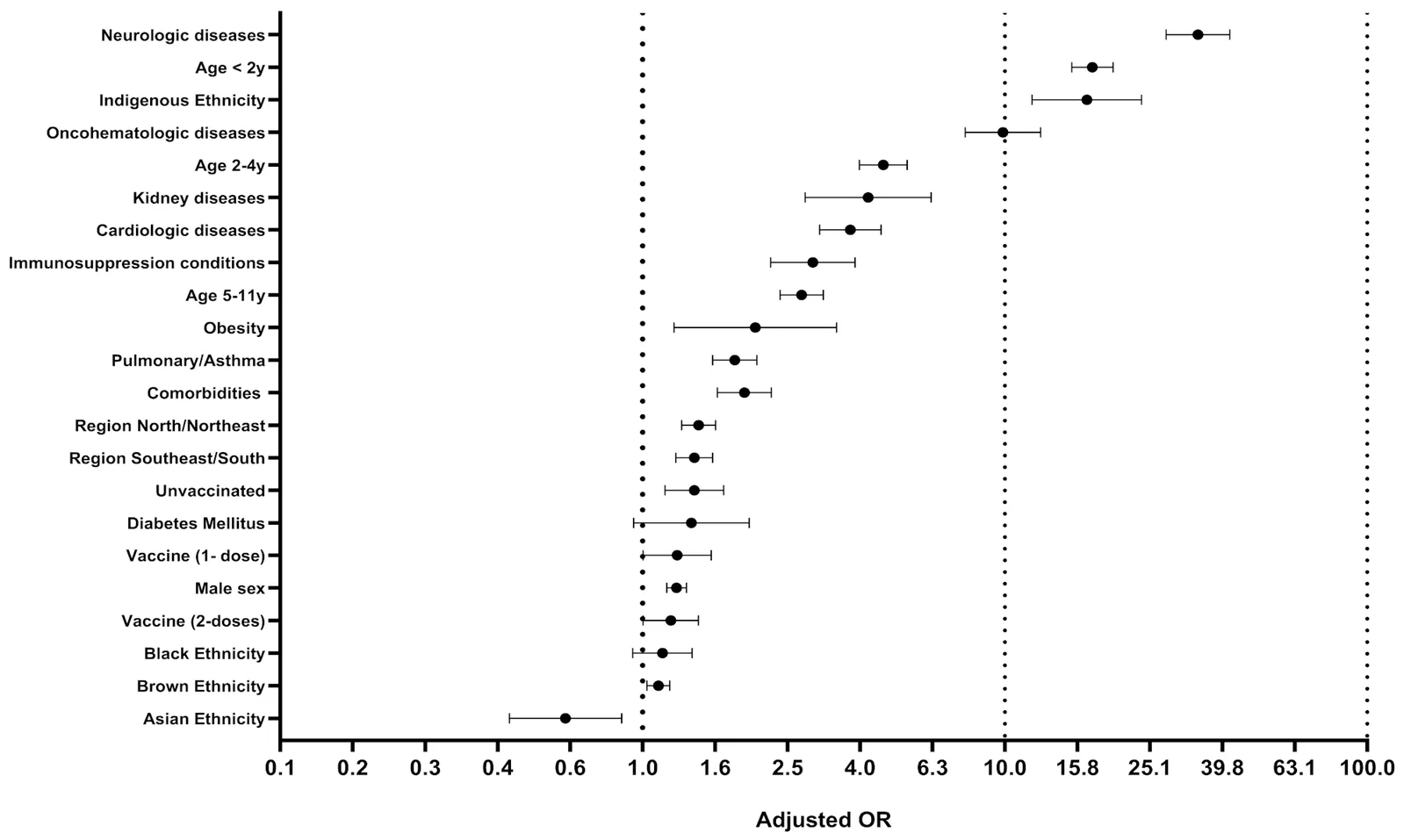
Risk factors for severe COVID-19. The figure illustrates the risk factors associated with an increased risk of severe COVID-19 disease in descending order. Circles denote adjusted odds ratios (aOR), while horizontal lines indicate 95% confidence intervals derived from the multivariable model for in-hospital 60-day mortality. The reference categories for each covariate are presented in Table 1. Note: The log-transformed x-axis ensures that aORs > 1.0 and their inverses < 1.0 are equidistant from the null, enabling a symmetrical visual comparison of protective and harmful effects.

**Figure 3:**
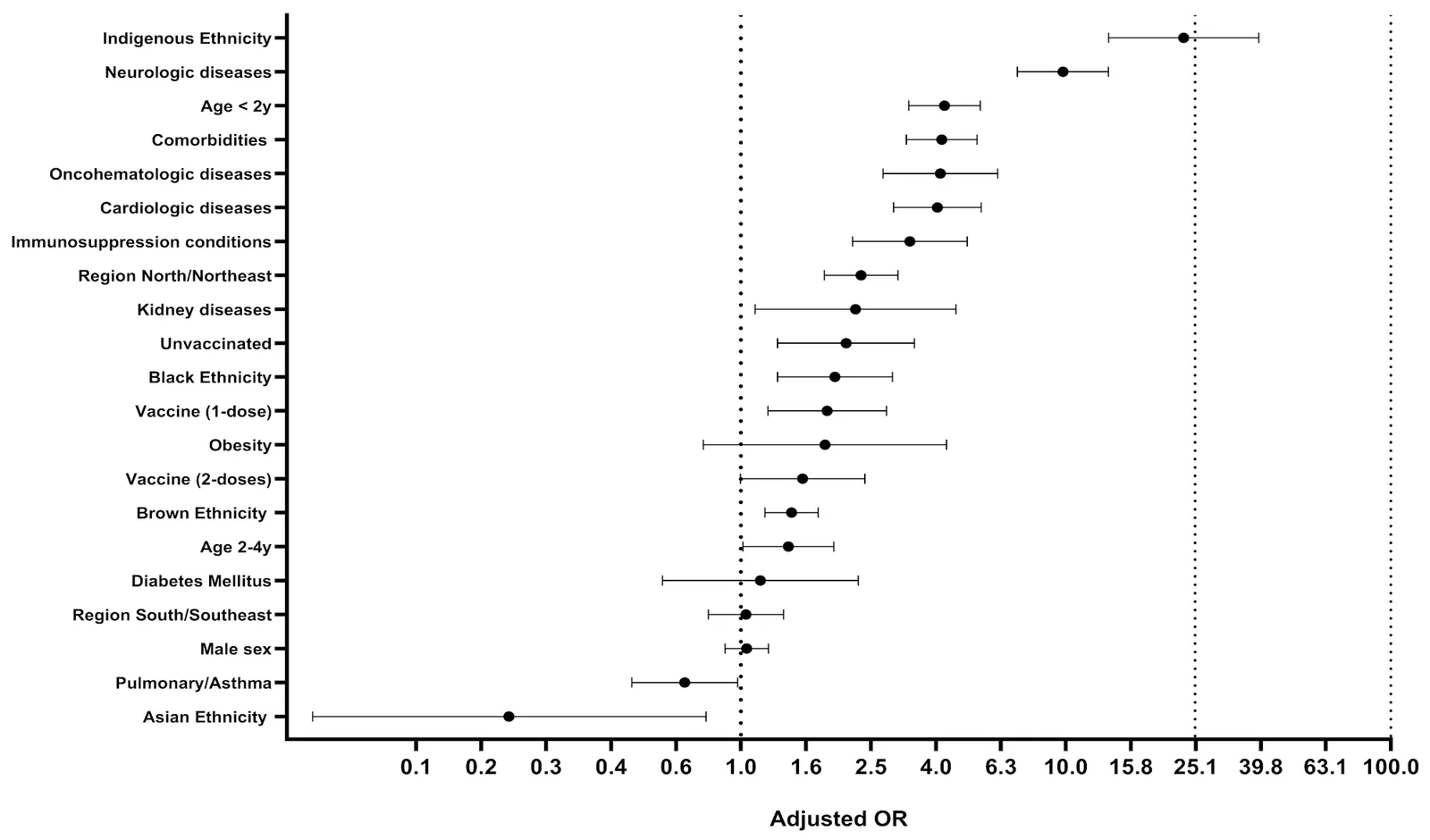
Risk factors for COVID-19-related death. The figure illustrates the risk factors associated with an increased risk of COVID-related death in descending order. Circles denote adjusted odds ratios (aOR), while horizontal lines indicate 95% confidence intervals derived from the multivariable model for in-hospital 60-day mortality. The reference categories for each covariate are presented in Table 1. Note: The log-transformed x-axis ensures that aORs > 1.0 and their inverses < 1.0 are equidistant from the null, enabling a symmetrical visual comparison of protective and harmful effects.

Vaccination was analyzed by dose count rather than by age-specific schedule completion because the vaccine platform was not available in the analytic dataset. Children who received three or more doses served as the reference category in the multivariable models; therefore, this category does not appear as a separate estimate in Figure 2 and Figure 3. Children whose vaccination status could not be determined (n = 96,668, 20.8%) were excluded from the vaccination-specific analyses.

### 2.3. Outcomes

The primary outcomes were (1) in-hospital mortality occurring in the first 60 days of hospitalization for laboratory-proven COVID-19 and (2) severe COVID-19, a composite endpoint defined as admission to the intensive care unit (ICU), need for mechanical ventilation, or death [28].

### 2.4. Statistical Analysis

Continuous variables were summarized using medians and interquartile ranges (IQRs) or means and standard deviations, as appropriate. Categorical variables were described using frequency and proportion. Groups were compared using the chi-square test (for proportions) and the Mann–Whitney U test (for medians).

Bivariate and multivariate regression analyses were used to ascertain the risk factors associated with COVID-19-related severe disease and mortality. Separate regression models were constructed for each of the outcomes. In these models, the outcomes (severe disease and mortality) served as dependent variables, whereas clinical and demographic data along with vaccination status (categorized as unvaccinated, one dose, two doses, and three or more doses) were included as independent variables. The clinical and demographic variables included in the model were age, sex, ethnicity, region of the country, and comorbidities. These variables were selected a priori as clinically and epidemiologically plausible confounders. Analyses were performed using the statistical software packages R (version 4.3.0, The R Foundation), SPSS (version 29), and STATA (version 18). Statistical significance was set at *p* < 0.05.

Ethical considerations. This study used de-identified publicly available data from the SIVEP-Gripe and e-SUS Notifica databases. The study protocol was approved by the Research Ethics Committee of the Federal University of Minas Gerais (Approval Number: 6.127.414 (19 June 2023). Following ethically agreed principles on anonymized open data, this analysis did not require an informed consent form in Brazil. This study was conducted and reported following the Strengthening the Reporting of Observational Studies in Epidemiology (STROBE) guidelines for cohort studies.

## 3. Results

### 3.1. Study Population and Clinical Outcomes

Of the 465,689 children and adolescents who met the study criteria, 18,784 (4.0%) were hospitalized, 5271 (1.1%) were admitted to an ICU, 1408 (0.3%) received mechanical ventilation, 847 (0.18%) died, and 5963 (1.3%) developed severe disease. In our cohort of 465,689 individuals, 194,463 were unvaccinated (41.8%), 35,072 had received one dose (7.5%), 99,607 had received two doses (21.4%), 39,879 had received three or more doses (8.6%), and for 96,668 individuals (20.8%), their vaccination status was unknown.

### 3.2. Factors Associated with Severe Disease

Table 1 presents the clinical profiles and risk factors for severe COVID-19. Patients with severe disease were significantly younger than those without severe disease (mean age 3.1 [SD 4.4] vs. 9.0 [SD 5.8] years). Children aged 0–1.9 years accounted for 64.7% (3855/5963) of all severe cases in the study. In bivariate analysis, the strongest risk factors for severe disease were the presence of three or more comorbidities (OR = 62.4, 95% CI: 45.7–85.1), Indigenous ethnicity (OR = 48.4, 95% CI: 35.6–65.8), and age under 2 years (OR = 22.5, 95% CI: 20.4–24.9). All major comorbidities were significantly associated with a higher risk, and unvaccinated children had more than four times higher odds of severe disease (OR = 4.6, 95% CI: 4.0–5.4) (Table 1).

**Table 1:** Factors associated with severe COVID-19 illness in children and adolescents with SARS-CoV-2 infection during the post-pandemic period (2023–2025).

Covariates *	Non-Severe COVID-19 (%)	Severe COVID-19 (%)	Unadjusted OR (95% CI)	*p*-Value
	459,726 (98.7)	5963 (1.3)		
Age (years)				
Mean (SD)	9.0 (5.8)	3.1 (4.4)	0.807 (0.802–0.812)	<0.001
Age group (years)				
12.0–17.9	190,203 (99.8)	423 (0.2)	1	<0.001
5.0–11.9	137,432 (99.3)	965 (0.7)	3.2 (2.8–3.5)	<0.001
2.0–4.9	55,209 (98.7)	720 (1.3)	5.9 (5.2–6.6)	<0.001
<2 y	76,882 (95.2)	3855 (4.8)	22.5 (20.5–24.9)	<0.001
Sex				
Male	223,191 (48.6)	3369 (56.5)	1	<0.001
Female	235,998 (51.4)	2594 (43.5)	1.4 (1.3–1.4)	<0.001
Region				
Southeast	236,759 (51.6)	3013 (50.5)	1	<0.001
South	66,617 (14.5)	768 (12.9)	0.90 (0.84–0.98)	0.015
Central-West	63,584 (13.8)	694 (11.6)	0.86 (0.79–0.93)	<0.001
Northeast	60,218 (13.1)	1122 (18.8)	1.51 (1.41–1.60)	<0.001
North	32,021 (7.0)	366 (6.1)	0.90 (0.80–1.00)	0.054
Ethnicity				
White	188,014 (51.6)	2414 (48.6)	1	<0.001
Brown	155,939 (42.8)	2301 (46.3)	1.1 (1.09–1.22)	<0.001
Black	9860 (2.7)	145 (2.9)	1.1 (0.97–1.36)	0.115
Asian	10,505 (2.9)	39 (0.8)	0.3 (0.21–0.40)	<0.001
Indigenous	108 (0.0)	67 (1.3)	48.4 (35.6–65.8)	<0.001
Signs/symptoms				
Fever	225,218 (49.0)	4063 (68.1)	2.2 (2.1–2.3)	<0.001
Cough	248,518 (54.1)	4087 (68.5)	1.8 (1.7–1.9)	<0.001
Dyspnea	36,591 (8.0)	3313 (55.6)	14.4 (13.7–15.2)	<0.001
Odynophagia	143,350 (31.2)	382 (6.4)	0.15 (0.14–0.17)	<0.001
Number of comorbidities				
None	435,482 (94.8)	4478 (75.1)	1	<0.001
1	22,771 (5.0)	1169 (19.6)	5.0 (4.78–5.3)	<0.001
2	843 (0.2)	250 (4.2)	28.8 (24.9–33.3)	<0.001
3 or more	103 (0.0)	66 (1.1)	62.4 (45.7–85.1)	<0.001
Major comorbidities				
Neurologic disorders	522 (0.1)	450 (7.5)	71.8 (63.1–81.7)	<0.001
Oncohematologic	355 (0.1)	234 (3.9)	52.8 (44.7–62.6)	<0.001
Cardiovascular	1455 (0.3)	320 (5.4)	17.9 (15.8–20.2)	<0.001
Kidney diseases	289 (0.1)	49 (0.8)	13.2 (9.7–17.8)	<0.001
Immunosuppression	1007 (0.2)	122 (2.0)	9.5 (7.9–11.6)	<0.001
Diabetes mellitus	763 (0.2)	52 (0.9)	5.3 (4.0–7.0)	<0.001
Pulmonary/Asthma	9629 (2.1)	418 (7.0)	3.5 (3.2–3.9)	<0.001
Obesity	563 (0.1)	26 (0.4)	3.6 (2.4–5.3)	<0.001
Vaccine doses				
Three or more	39,693 (99.5)	186 (0.5)	1	
Two	98,802 (99.2)	805 (0.8)	1.7 (1.5–2.0)	<0.001
One	34,791 (99.2)	281 (0.8)	1.7 (1.4–2.1)	<0.001
None	190,348 (97.9)	4115 (2.1)	4.6 (3.4–5.3)	<0.001

* Available data for covariates with missing data: Sex (n = 465,152), Region (n = 465,162), Ethnicity (n = 369,392), and Vaccine doses (n = 352,096).

Figure 2 ranks the adjusted odds ratios (aORs) for covariates associated with severe disease in descending order, based on the binary regression analysis. Among clinical factors, neurological disease was the strongest independent risk factor (aOR = 34.1, 95% CI: 27.9–41.8), while Indigenous ethnicity was the strongest demographic factor (aOR = 16.8, 95% CI: 11.9–23.8). Age showed an inverse pattern of association: compared to the reference group (12–17.9 years), children aged <2 years (aOR = 17.4, 95% CI: 15.3–19.9), 2–4 years (aOR = 4.4, 95% CI: 3.8–5.2), and 5–11 years (aOR = 2.6, 95% CI: 2.3–3.0) all had a significantly higher risk of severe disease.

Most major comorbidities were independent predictors, with the exception of diabetes mellitus (aOR = 1.37, 95% CI: 0.94–1.71, *p* = 0.09). Compared to the Central-West region, the North/Northeast (aOR = 1.43, 95% CI: 1.23–1.60) and South/Southeast (aOR = 1.39, 95% CI: 1.23–1.56) regions had similar increased odds of severe disease. Compared to White children, Brown ethnicity was associated with a modestly higher risk (aOR = 1.10, 95% CI: 1.03–1.18), while Black ethnicity did not differ significantly (aOR = 1.13, 95% CI: 0.94–1.37). Asian ethnicity was protective against severe disease (aOR = 0.61, 95% CI: 0.43–0.87). Vaccination remained independently protective; relative to children receiving three or more doses, unvaccinated children had 39% higher odds of severe disease (aOR = 1.39, 95% CI: 1.15–1.67). Receiving only one or two doses also conferred a higher risk than the complete vaccination schedule (Figure 2).

Table 2 presents the risk factors for COVID-19-related deaths. In the bivariate analysis, each one-year increase in age was associated with a 13% reduction in mortality risk (OR = 0.87, 95% CI: 0.86–0.88). Younger age groups showed a progressively higher risk of death than adolescents. Other covariates associated with increased odds of death included male sex, residence in the South, Northeast, or North regions, non-White ethnicities, the presence of fever or dyspnea at baseline, and the presence of comorbidities (showing a dose–response pattern). Fewer than three vaccine doses were incrementally associated with death: two doses (OR = 1.69, 95% CI: 1.19–2.55), one dose (OR = 2.28, 95% CI: 1.45–3.58), and no doses (OR = 4.35, 95% CI: 2.91–6.21) (Table 2).

Figure 3 shows the adjusted associations from the multivariable binary logistic regression analysis. Indigenous ethnicity was the strongest independent risk factor for death (aOR = 23.5, 95% CI: 13.5–39.3), followed by neurologic disease (aOR = 9.8, 95% CI: 7.1–13.5) and age < 2 years (aOR = 4.2, 95% CI: 3.3–5.4). Other chronic conditions strongly associated with death included oncohematologic disease (aOR = 4.1, 95% CI: 2.7–6.1), cardiologic disease (aOR = 4.0, 95% CI: 2.9–5.4), and kidney disease (aOR = 2.2, 95% CI: 1.1–4.6). Obesity was not significantly associated with mortality in the multivariable model (aOR = 1.8, 95% CI: 0.76–4.3). Unvaccinated children had twice the risk of death compared to those who received three doses (aOR = 2.1, 95% CI: 1.3–3.5, *p* < 0.001).

### 3.3. Contrasting Patterns Between Severe Disease and Mortality

Several factors showed divergent associations with severe disease and mortality. While the South/Southeast (aOR = 1.39) and North/Northeast (aOR = 1.43) regions had similar effect sizes for severe disease, the odds of death were more than double for children in the North/Northeast (aOR = 2.3, 95% CI: 1.8–3.0) compared to the Central-West reference; in contrast, children from the South/Southeast had no significantly elevated mortality risk (aOR = 1.03, 95% CI: 0.79–1.35). Regarding ethnicity, Brown and Black children had similar or modestly higher risk of severe illness but significantly higher mortality than White children (Black: aOR = 1.95, 95% CI: 1.29–2.92; Brown: aOR = 1.43, 95% CI: 1.18–1.73). Asian ethnicity remained strongly protective against mortality (aOR = 0.19, 95% CI: 0.05–0.78). Male sex was independently associated with severe disease (aOR = 1.23, 95% CI: 1.15–1.31) but not with mortality (aOR = 1.04, 95% CI: 0.89–1.21). Notably, asthma/chronic pulmonary disease showed an inverse association, appearing protective against mortality (aOR = 0.67, 95% CI: 0.46–0.98, *p* = 0.04).

## 4. Discussion

### 4.1. Key Findings

In a large-scale Brazilian cohort of 465,689 pediatric patients with laboratory-confirmed COVID-19 in the post-pandemic period, the most vulnerable subgroups for severe outcomes were young children (especially infants), those with major comorbidities, and Indigenous children. Notably, incomplete vaccination (<three doses) was associated with significantly increased vulnerability. Unvaccinated children had an independent twofold odds of death (aOR = 2.1; 95% CI, 1.3–3.5) and approximately 40% higher odds of severe COVID-19 than those who received ≥ three doses of vaccine.

### 4.2. Findings in Context

#### 4.2.1. Severity and Mortality Rates

During the post-pandemic period, hospitalization (4% vs. 2.1%) and ICU admission (1.1% vs. 0.48%) rates were higher than those during the pandemic, but the overall mortality rate (0.18%) remained similar to the pandemic-era rate of 0.17% [11]. Risk factors for severe outcomes, including comorbidities and sociodemographic characteristics (ethnicity and geographic region), remained broadly similar to those identified during the pandemic. However, in the post-pandemic period, young age and unvaccinated status emerged as particularly prominent risk factors for severe outcomes.

#### 4.2.2. Age as a Risk Factor

Age strongly influenced rates of severe COVID-19 and death, with risk increasing steadily as age decreased; infants faced the highest odds. This pattern contrasts with early pandemic data, when our group and others reported a U-shaped curve with elevated risks at both age extremes (infants and adolescents) [5,7]. Crucially, these earlier findings originated from a period before pediatric vaccination was available. Contemporary studies indicate that young, unvaccinated children are now at the highest risk [20,29]. Although COVID-19 vaccines are effective in preventing severe disease in children [26,30,31,32,33,34,35], vaccine coverage among children, particularly the youngest, remains suboptimal compared to adults [29,36]. Brazilian data also show incomplete and unequal pediatric coverage: among children aged 5–11 years, first- and second-dose coverage reached 69.5% and 46.1%, respectively, by December 2022, with lower coverage in municipalities with lower human development and in the North and Northeast regions [37]. In our cohort, 63.5% of adolescents had received two or three doses of the vaccine, whereas only 8.5% of children under the age of four had completed the vaccination schedule. This discrepancy may partially account for the heightened vulnerability observed among younger children.

#### 4.2.3. Comorbidities

The presence of an underlying chronic medical condition was a primary factor for severe outcomes, consistent with established evidence that comorbidities increase the risk of critical COVID-19 outcomes [28,38]. Our previous pandemic-era analyses consistently demonstrated a strong, incremental association between the number of comorbidities and severe illness [7,11], a dose–response relationship confirmed by systematic reviews [5,10]. Our findings extend this pattern to the post-pandemic period.

Among the comorbidities, neurological disorders were most strongly associated with severe outcomes. This aligns with systematic reviews identifying cardiac and neurological conditions as conferring the highest risk for critical care or death [5,10]. Children with chronic neurological disorders often have multimorbidity and are at the greatest risk of severe COVID-19 [39]. Cardiovascular, oncohematologic, and kidney diseases were also strongly associated with severe outcomes and death, replicating the findings of systematic reviews [5,10]. In contrast, obesity and diabetes mellitus were strong predictors in the bivariate analysis but lost statistical significance for mortality in the multivariable model. Although Aparicio et al. [5] reported twofold higher odds of critical disease in children with these conditions, and our own pandemic-era analyses showed increased mortality risk for each [40,41], the current cohort revealed that nearly all children with obesity or diabetes had at least one additional comorbidity and tended to be older. This clustering of risk factors likely weakened the independent association in the multivariable analysis, rather than indicating a true absence of risk in the population. Our analysis revealed that children with asthma or pulmonary disease exhibited a significantly lower risk of mortality related to COVID-19. Cohort studies have yielded inconsistent findings regarding the risk of mortality from COVID-19 in children and adults with asthma [42,43]. In our prior study conducted during the pandemic, it was observed that children with asthma experienced a 60% reduction in the hazard of death compared to their healthy counterparts [44]. Several mechanisms have been proposed to explain the milder clinical course of COVID-19 in patients with asthma. These include an immune-dominant T helper 2 environment that mitigates hyperinflammation [45], the presence of protective genes [46], and decreased expression of angiotensin-converting enzyme 2 (ACE2) in the lungs of children with atopy [47]. Furthermore, the use of inhaled corticosteroids (ICS) may exert anti-inflammatory and antiviral effects, potentially reducing the severity of COVID-19 [48].

#### 4.2.4. Social Determinants of Health

Our study highlights the intricate relationship between SDOH and COVID-19 outcomes in the post-pandemic period in Brazil. During the pandemic, social and racial inequalities emerged as risk factors in both high- and low-to-middle-income countries [49,50,51,52]. In our analysis, comparing macroregions with distinct historical, socioeconomic, and healthcare access characteristics (using the Central-West region as a reference), we observed that children from both the Southeast/South and Northeast/North regions had similar higher risks of severe COVID-19 than the reference. However, children residing in the socioeconomically deprived Northeast/North regions had twofold higher odds of fatal outcomes, whereas those from the wealthier Southeast/South regions had no significant odds of mortality compared with the Central-West region. Similar findings have been reported for adult, pediatric, and pregnant populations in Brazil during the pandemic [53,54,55,56]. Santos et al. [57] identified seven spatial clusters of COVID-19 mortality: four in the Northeast and three in the North regions.

Ethnicity is intrinsically related to the SDOH. Indigenous children had the highest risk of both severe disease and COVID-19-related death in the post-pandemic period, consistent with our previous pandemic-era studies [7,11]. Structural factors, such as poverty, geographic isolation, and systematic discrimination, place Indigenous populations at an increased risk of emerging infectious diseases [58,59]. Additionally, in our analysis, Brown/Black ethnicities, historically marginalized populations in Brazil, had similar higher risks of severe illness but a significantly higher mortality risk than White children. In contrast, Asian ethnicity was a strong protective factor for severe outcomes. Taken together, our data underscore that children from poor, neglected regions and historically discriminated ethnicities still carry the highest burden of COVID-19 in the post-pandemic period in Brazil. These findings highlight the mechanisms through which poverty and discrimination affect outcomes, including structural inequalities, greater pre-existing health challenges, reduced healthcare access, lower-quality housing, and low vaccination coverage.

#### 4.2.5. Vaccination

Vaccination is arguably the only modifiable intervention that can be implemented in the short term to reduce the severity of COVID-19 outcomes. Our analysis demonstrated that the lack of a complete COVID-19 vaccination schedule increased the risk of severe illness and death in the post-pandemic era. In a clear dose–response manner, unvaccinated children had roughly fourfold odds of severe disease and mortality compared with children with a complete vaccination schedule. After adjustment, unvaccinated children had double the risk of death, while those with one or two doses had 80% and 50% higher risks, respectively, than children with at least three doses. Notably, among children who experienced severe outcomes, approximately 76% were unvaccinated, and only approximately 3.5% had received three or more doses.

Post-pandemic vaccination data for children are scarce. Between 2023 and 2024, Tartof et al. [34] reported an estimated vaccine effectiveness of the BNT162b2 XBB vaccine for children aged 5–17 years of 65% (95% CI, 36–81%) against hospital admission and emergency department/urgent care visits [31]. Recently, we showed that in the post-pandemic period, the estimated VE against death remained high, reaching 89.4% (95% CI, 29.8–98.7%) and 75.8% (95% CI, 36.4–95.7%) for children with and without comorbidities, respectively [26].

### 4.3. Strengths and Limitations

#### 4.3.1. Strengths

The primary strengths of this study are its scale, representativeness, and temporal scope. By combining data from e-SUS Notifica and SIVEP-Gripe, we captured both non-hospitalized and hospitalized cases across the entire Brazilian territory over a 30-month post-pandemic window, which, to our knowledge, represents one of the largest available pediatric COVID-19 datasets in the post-pandemic era.

#### 4.3.2. Limitations

This study has several limitations that must be acknowledged. First, we utilized large-scale administrative healthcare datasets. While these offer substantial sample sizes and population coverage, they have inherent limitations, including missing data and insufficient granularity for relevant confounding factors, such as social determinants and detailed clinical data (e.g., laboratory and imaging results) [60]. Nevertheless, such datasets provide a robust perspective on risk factors that would be logistically impractical to achieve using traditional prospective cohorts. Second, missing data are an inherent challenge in administrative database studies [61]. Vaccination status was unknown for approximately 21% of our cohort, which may have introduced misclassification bias that could have underestimated the protective effects of the vaccine. An additional limitation is that the vaccine platform was unavailable for analysis, preventing the mapping of dose count onto the completion of the age-appropriate schedule. Consequently, the category of three or more doses includes children who completed the primary series and those who received booster doses, leading to clinical non-equivalence across age groups. This issue may introduce bias into the vaccination-outcome association by underestimating the effect size, as a clinically heterogeneous reference group is likely to dilute, rather than exaggerate, the observed protective effects. Third, the determination of comorbidity relied on the completeness of fields filled by reporting clinicians, which may have resulted in underreporting of certain conditions. In this context, imputing missing information as the absence of a condition could have led to misclassification of comorbidity status. As a result, the bias introduced would likely attenuate (bias toward the null) rather than exaggerate the observed associations between comorbidities and outcomes, suggesting that our estimates may be conservative in nature. Moreover, the non-coded category of “neurological disease” (heterogeneous by nature) makes it difficult to determine which conditions are driving the strong observed association. Additionally, as stated above, official Brazilian datasets lack granularity regarding specific preexisting conditions. Therefore, information regarding specific comorbidities for each of the systems was not available in the datasets, and consequently, their classification of severity was also not available. Unfortunately, these database characteristics precluded a more refined analysis of the comorbidity data. Finally, the surveillance systems do not capture post-acute sequelae, limiting our conclusions to acute-phase outcomes.

The databases lacked information on family-level income and direct measures of access to advanced care facilities. Consequently, we were unable to adjust the models for unobserved confounders, such as household income and direct healthcare access, owing to the absence of these variables. Consequently, it was not possible to disentangle the effects of ethnicity, geography, socioeconomic conditions, and healthcare access at a more detailed level. Additionally, we could not determine whether the increased risk among Indigenous children varied across specific territories or Indigenous populations in Brazil. Furthermore, the regions were grouped based on their social development status to maintain statistical power. However, this approach may have obscured finer geographic variations, suggesting that future research linking outcomes to state- or municipality-level indicators could facilitate a more detailed geographic analysis. Addressing these questions necessitates linking municipal socioeconomic indicators, geocoded residences, health service availability, and comprehensive information on Indigenous communities.

Future research should extend the findings of our study by examining the linkage of the adverse outcomes with municipal socioeconomic and Indigenous community-level data, prospective vaccine-platform-specific studies, and long-term/post-acute outcome tracking. In addition, the development and equitable availability of affordable antiviral therapies, in alignment with the objectives of the UN Sustainable Development Goals, is an important complementary strategy alongside vaccination for pandemic preparedness [62,63]. As current surveillance tools lack the capability to stratify the severity of comorbidities such as diabetes, asthma, and obesity, future research should focus on refining risk stratification for these chronic conditions. This should be conducted in large cohort studies that feature more comprehensive database linkages.

#### 4.3.3. Study Implications

The findings of this study have considerable implications for public health. Our results identified specific pediatric groups as high-priority targets for booster vaccination. Notably, the highest risk of severe outcomes has now shifted to younger age groups, which demonstrate lower complete vaccination coverage than adolescents [37,64,65]. Consequently, public health initiatives should focus on enhancing vaccination coverage among these vulnerable groups. Furthermore, empirical evidence derived from comprehensive real-world data from a continental nation such as Brazil may contribute to reducing COVID-19 vaccine hesitancy among parents and caregivers, thereby protecting children at greatest risk [66].

## 5. Conclusions

Our analysis of a substantial pediatric cohort identified that the principal risk factors for severe COVID-19 outcomes and mortality within the first 60 days of hospitalization in the post-pandemic era were largely consistent with those observed during the pandemic, albeit with some differences. The influence of social determinants of health (SDOH) on severe COVID-related outcomes, which disproportionately affected socioeconomically disadvantaged individuals during the pandemic, persists in affecting children and adolescents three years following the end of the public health emergency. Notably, residing in vulnerable areas and belonging to non-White ethnic groups, particularly Indigenous populations, heightened the risk of COVID-related mortality. Additionally, the risk of severe illness and mortality has increasingly shifted towards younger children, especially infants. Therefore, public health initiatives should prioritize enhancing vaccination coverage among these vulnerable populations and pregnant women to protect young infants for whom vaccines are not yet available. All significant chronic underlying conditions continued to be strongly associated with severe outcomes, with neurological disorders presenting the highest risk. Finally, unvaccinated status emerged as an independent predictor of severe outcomes, demonstrating a clear dose–response relationship.

## Figures and Tables

**Table 2:** Factors associated with COVID-19-related death in children and adolescents with SARS-CoV-2 infection during the post-pandemic period (2023–2025).

Covariates *	Survival (%)	Death (%)	Unadjusted OR (95% CI)	*p*-Value
	464,842 (99.8)	847 (0.2)		
Age (years)				
Mean (SD)	8.94 (5.8)	4.56 (5.6)	0.87 (0.86–0.88)	<0.001
Age group (years)				
12–17.9	190,485 (99.9)	141 (0.1)	1	1
5–11.9	138,253 (99.9)	144 (0.1)	1.4 (1.1–1.8)	0.04
2–4.9	55,833 (99.8)	96 (0.2)	2.3 (1.8–3.0)	<0.001
<2 y	80,271 (99.4)	466 (0.6)	7.8 (6.5–9.5)	<0.001
Sex				
Female	238,467 (99.8)	402 (0.2)	1	1
Male	226,365 (99.8)	445 (0.2)	1.2 (1.02–1.3)	0.026
Region				
Southeast	239,490 (51.6)	282 (33.3)	1	
South	67,281 (14.5)	104 (12.3)	1.3 (1.05–1.6)	0.017
Central-West	64,187 (13.8)	91 (10.7)	1.2 (0.9–1.5)	0.122
Northeast	61,125 (13.2)	215 (25.4)	2.9 (2.5–3.6)	<0.001
North	32,232 (6.9)	155 (18.3)	4.0 (3.4–4.9)	<0.001
Ethnicity				
White	190,173 (51.6)	255 (34.1)	1	
Brown	157,804 (42.8)	436 (58.4)	2.0 (1.8–2.4)	<0.001
Black	9975 (2.7)	30 (4.0)	2.2 (1.5–3.3)	<0.001
Asian	10,541 (2.9)	3 (0.4)	0.21 (0.07–0.66)	0.008
Indigenous	152 (0.0)	23 (3.1)	113.0 (71.7–178.2)	<0.001
Signs/symptoms				
Fever	228,743 (49.3)	538 (63.5)	1.8 (1.6–2.1)	<0.001
Cough	252,136 (54.3)	469 (55.4)	1.0 (0.91–1.2)	0.533
Dyspnea	39,429 (8.5)	476 (56.2)	13.8 (12.1–15.8)	<0.001
Odynophagia	143,628 (30.9)	104 (12.3)	0.31 (0.25–0.38)	<0.001
Comorbidities				
None	439,413 (94.6)	547 (64.6)	1	
1	23,726 (5.1)	214 (25.3)	7.2 (6.2–8.5)	<0.001
2	1037 (0.2)	56 (6.6)	43.4 (32.8–57.6)	<0.001
3 or more	139 (0.0)	30 (3.5)	173.6 (115.9–259.8)	<0.001
Major comorbidities				
Neurologic	890 (0.2)	82 (9.7)	55.9 (44.1–70.8)	<0.001
Oncohematologic	543 (0.1)	46 (5.4)	49.1 (36.0–66.8)	<0.001
Cardiovascular	1681 (0.4)	94 (11.1)	34.4 (27.6–42.8)	<0.001
Immunosuppression	1085 (0.2)	44 (5.2)	23.4 (17.2–31.9)	<0.001
Kidney diseases	326 (0.1)	12 (1.4)	20.4 (11.4–36.5)	<0.001
Diabetes mellitus	802 (0.2)	13 (1.5)	9.0 (5.3–15.6)	<0.001
Obesity	580 (0.1)	9 (1.1)	8.6 (4.4–16.6)	<0.001
Pulmonary/Asthma	10,000 (2.2)	47 (5.5)	2.7 (1.9–3.6)	<0.001
Vaccine schedule				
Three or more	39,851 (99.9)	28 (0.1)	1	
Two	99,489 (99.9)	118 (0.1)	1.7 (1.2–2.5)	0.013
One	35,016 (99.8)	56 (0.2)	2.3 (1.4–3.6)	<0.001
None	193,884 (99.7)	579 (0.3)	4.3 (2.9–6.2)	<0.001

* Available data for covariates with missing data: Sex (n = 465,152), Region (n = 465,162), Ethnicity (n = 369,392), and Vaccine doses (n = 352,096).

## Data Availability

All SIVEP-Gripe and e-SUS Notifica data are publicly available at https://dados.gov.br/dados/conjuntos-dados/srag-2021-e-2022 (accessed on 10 July 2025). The analysis code and research data supporting this publication are available upon request from the corresponding author (Eduardo A. Oliveira; eduolive812@gmail.com).

## Abbreviations

The following abbreviations are used in this manuscript:

COVID-19	Coronavirus disease 2019
SARS-CoV-2	Severe acute respiratory syndrome coronavirus 2
SIVEP-Gripe	Surveillance Information System for Influenza
OR	Odds ratio
aOR	Adjusted odds ratio
CI	Confidence interval
ICU	Intensive care unit
SDOH	Social determinants of health
e-SUS Notifica	Brazilian outpatient COVID-19 notification system
IBGE	Brazilian Institute of Geography and Statistics
STROBE	Strengthening the Reporting of Observational Studies in Epidemiology
Anvisa	Brazilian National Health Surveillance Agency
PNI	Brazilian National Immunization Program
VE	Vaccine effectiveness
SD	Standard deviation
SUS	Brazilian Unified Health System
